# Mitogenomics of the Old World monkey tribe Papionini

**DOI:** 10.1186/s12862-014-0176-1

**Published:** 2014-09-04

**Authors:** Rasmus Liedigk, Christian Roos, Markus Brameier, Dietmar Zinner

**Affiliations:** Primate Genetics Laboratory, German Primate Center, Leibniz Institute for Primate Research, Kellnerweg 4, 37077 Göttingen, Germany; Gene Bank of Primates, German Primate Center, Leibniz Institute for Primate Research, Kellnerweg 4, 37077 Göttingen, Germany; Cognitive Ethology Laboratory, German Primate Center, Leibniz Institute for Primate Research, Kellnerweg 4, 37077 Göttingen, Germany

**Keywords:** Phylogeny, Divergence ages, mtDNA, Primates, Macaques, Baboons

## Abstract

**Background:**

The evolutionary history of the Old World monkey tribe Papionini comprising the genera *Macaca*, *Mandrillus*, *Cercocebus*, *Lophocebus*, *Theropithecus*, *Rungwecebus* and *Papio* is still matter of debate. Although the African Papionini (subtribe Papionina) are generally considered to be the sister lineage to the Asian Papionini (subtribe Macacina), previous studies based on morphological data, nuclear or mitochondrial sequences have shown contradictory phylogenetic relationships among and within both subtribes. To further elucidate the phylogenetic relationships among papionins and to estimate divergence ages we generated mitochondrial genome data and combined them with previously published sequences.

**Results:**

Our mitochondrial gene tree comprises 33 papionins representing all genera of the tribe except *Rungwecebus*. In contrast to most previous studies, the obtained phylogeny suggests a division of the Papionini into three main mitochondrial clades with similar ages: 1) *Papio*, *Theropithecus*, *Lophocebus*; 2) *Mandrillus*, *Cercocebus*; and 3) *Macaca*; the *Mandrillus* + *Cercocebus* clade appears to be more closely related to *Macaca* than to the other African Papionini. Further, we find paraphyletic relationships within the *Mandrillus* + *Cercocebus* clade as well as in *Papio*. Relationships among *Theropithecus*, *Lophocebus* and *Papio* remain unresolved. Divergence ages reveal initial splits within the three mitochondrial clades around the Miocene/Pliocene boundary and differentiation of *Macaca* species groups occurred on a similar time scale as those found between genera of the subtribe Papionina.

**Conclusion:**

Due to the largely well-resolved mitochondrial phylogeny, our study provides new insights into the evolutionary history of the Papionini. Results show some contradictory relationships in comparison to previous analyses, notably the paraphyly within the *Cercocebus* + *Mandrillus* clade and three instead of only two major mitochondrial clades. Divergence ages among species groups of macaques are similar to those among African Papionini genera, suggesting that diversification of the mitochondrial genome is of a similar magnitude in both subtribes. However, since our mitochondrial tree represents just a single gene tree that most likely does not reflect the true species tree, extensive nuclear sequence data is required to illuminate the true species phylogeny of papionins and to trace possible ancient hybridization events among lineages.

**Electronic supplementary material:**

The online version of this article (doi:10.1186/s12862-014-0176-1) contains supplementary material, which is available to authorized users.

## Background

It is well recognized that mitochondrial (mtDNA) phylogenies are not necessarily congruent with the phylogeny of the respective taxa or phylogenies based on a set of nuclear genes (e.g. [1–3]). Reasons for the incongruence are manifold, e.g., different inheritance pathways, divergent selection pressures, and most prominent, incomplete lineage sorting and horizontal gene flow (e.g. [4,5]). On the other hand, if mtDNA and nuclear (nDNA) phylogenies are congruent this could be a strong indication that the single underlying gene tree is congruent with the species tree. Furthermore, in many species analyses of mtDNA relationships provide a better spatial resolution, thus contributing to phylogeographical inferences [3,6]. Therefore, analyses of both, mtDNA and nDNA, are necessary for a comprehensive understanding of the evolutionary history of taxa and for a robust reconstruction of complex phylogenies.

Among primates, incongruences are reported for several taxa within the Old World monkey tribe Papionini (e.g. [7–14]). The Papionini tribe diverged from its sister lineage, the Cercopithecini, around 11.5 million years ago (Ma) [15] and comprises the subtribe Papionina, with the genera *Papio*, *Mandrillus*, *Theropithecus*, *Cercocebus*, *Rungwecebus* and *Lophocebus*, and the subtribe Macacina, with the genus *Macaca* [16]. While all available nDNA data and respective gene trees are congruent and strongly support this division [15,17,18], recent studies applying mtDNA genome data suggest the *Mandrillus + Cercocebus* clade to be closer related to *Macaca* [19,20], thus indicating paraphyly of Papionina in the mtDNA gene tree.

The African origin of the tribe is broadly accepted [16,21–25] and the fossil record indicates a Late Miocene dispersal out of Africa into Eurasia for some lineages. Remains of macaques have been found in southern, western and central Europe [26,27], whereas fossil macaques from Asia are documented but rather scarce [26]. Fossils of *Theropithecus* have been recovered from the Iberian Peninsula as well as from India [28–34]. Today the six genera of Papionina are found exclusively on the African continent, with the exception of the hamadryas baboon, which occurs in both northeastern Africa and the southwestern Arabian Peninsula [16,25]. In contrast, members of the subtribe Macacina are distributed over large regions of South, Southeast and East Asia with the exception of Barbary macaques, which are found in Northwest Africa. Based on morphological characters, the subtribe Papionina is divided into six relatively heterogeneous genera, while the Asian lineage consists of only one highly speciose genus (*Macaca*), which is divided into several species groups [16,23,26,35].

The tribe comprises 45 species [36], exhibiting a great variety of morphologies from more slender representatives like the crested mangabeys to more robust forms like baboons, mandrills and drills. The genus *Macaca* is divided into species groups, but the number and the composition of these species groups have been a matter of debate for decades [23,26,35]. Based on the morphology of male genitals Fooden [35] proposed four species groups comprising a *M. silenus-M. sylvanus*, a *M. fascicularis*, a *M. arctoides* and a *M. sinica* group, with a total of 19 species. Delson [26] also proposed four species groups but moved *M. arctoides* into the *M. sinica* group and separated *M. sylvanus* from the *M. silenus* lineage into its own group. Combining morphological and genetic data, Groves [23] proposed a classification into six species groups with a total of 20 species: (1) the monotypic *M. sylvanus* group, (2) the *M. nemestrina* group, (3) the Sulawesi group, (4) the *M. fascicularis* group, (5) the *M. mulatta* group and (6) the *M. sinica* group. In the most recent classification the genus *Macaca* consists of 22 species, in seven species groups [16], among them three monotypic species groups: (1) *M. sylvanus* group, (2) *M. arctoides* group and (3) *M. fascicularis* group, and four polytypic groups: (4) Sulawesi group, (5) *M. mulatta* group, (6) *M. sinica* group and (7) *M. silenus* group. Although the monophyly of the macaques was confirmed in several studies [23,26,35,37,38], relationships among and within the species groups are still disputed [37–40].

Similarly, within the African Papionina, relationships among genera and species are only partly resolved [41]. Findings based on morphological traits were often discordant with results from molecular studies. While early morphological analyses supported the monophyly of the mangabeys [42,43], more recent morphological [44–46] and molecular studies [17,47,48] suggested diphyly of mangabeys, with *Lophocebus* clustering with *Papio* and *Theropithecus*, while *Cercocebus* forms a clade with *Mandrillus*. The kipunji (*Rungwecebus kipunji*), earlier described as a member of *Lophocebus* [49], was recently placed in its own genus [50]. Subsequent genetic studies confirmed the diphyly of *Lophocebus* and *Cercocebus*, and in addition showed a close relationship of *Rungwecebus* to *Papio* [10,50,51]. Concerning *Papio*, genetic analyses revealed seven well-supported mtDNA haplogroups, but these were not congruent with the six recognized species of the genus [11,42,52–54]. Likewise, for the *Mandrillus + Cercocebus* clade a mtDNA study indicated paraphyly of *Cercocebus* with at least one species (*C. torquatus*) being more closely related to *Mandrillus* than to its congenerics [12], while nuclear gene trees suggest reciprocal monophyly of both genera [14,15]. Previous morphological studies noted some similarities between *Mandrillus*, *Cercocebus* and *Macaca*. Fleagle and McGraw [45,55] studied postcranial features of *Mandrillus*, *Cercocebus*, *Lophocebus* and *Papio* and compared them with respective data of one macaque species (*M. nemestrina*). Most characters of *Mandrillus* and *Cercocebus* did not differ from those of *M. nemestrina*, and were therefore interpreted to be primitive among papionins, whereas just one of the investigated traits in *M. nemestrina* did not differ from that of *Lophocebus*, *Papio* and *Theropithecus* [45,55]. Furthermore, although it is widely accepted that *Lophocebus* and *Theropithecus* cluster together with a clade consisting of *Papio* and *Rungwecebus*, the branching pattern among these lineages is unresolved [14,19,20,56].

It has recently been shown that the use of complete mtDNA genome sequences provide better statistical support in phylogenetic reconstructions when compared to analyses based on single genes or partial genomes (e.g. [57–60]). In our study we generated new mtDNA genome data of *Macaca* species and combined it with respective data of other Papionini from GenBank to reconstruct a robust mtDNA gene tree of papionin primates and to estimate respective divergence ages. We were particularly interested to obtain further information concerning the branching pattern among papionin genera and among all seven species groups of the genus *Macaca* and to provide comprehensive data for further comparative molecular studies.

## Results

We sequenced complete mtDNA genomes from eight macaques representing all seven macaque species groups: *M. sylvanus* – *M. sylvanus* group, *M. silenus* – *M. silenus* group*, M. tonkeana* – Sulawesi group, *M. thibetana* – *M. sinica* group, *M. mulatta*/China and *M. mulatta*/India – *M. mulatta* group, *M. fascicularis*/Vietnam – *M. fascicularis* group, and *M. arctoides – M. arctoides* group. A BLAST-search in GenBank showed that our newly generated sequences matched almost perfectly with available orthologs. The full-length genome sequences consisted of 13 protein-coding genes, 2 rRNA genes, 22 tRNA genes and the control region. The initial alignment comprised 38 sequences and had a length of 16,966 base pairs (bp). After indels and poorly aligned positions were removed the alignment comprised 15,685 bp including 6,986 informative sites. The alignment is available for download (Additional file 1 [61]).

**Figure 1:**
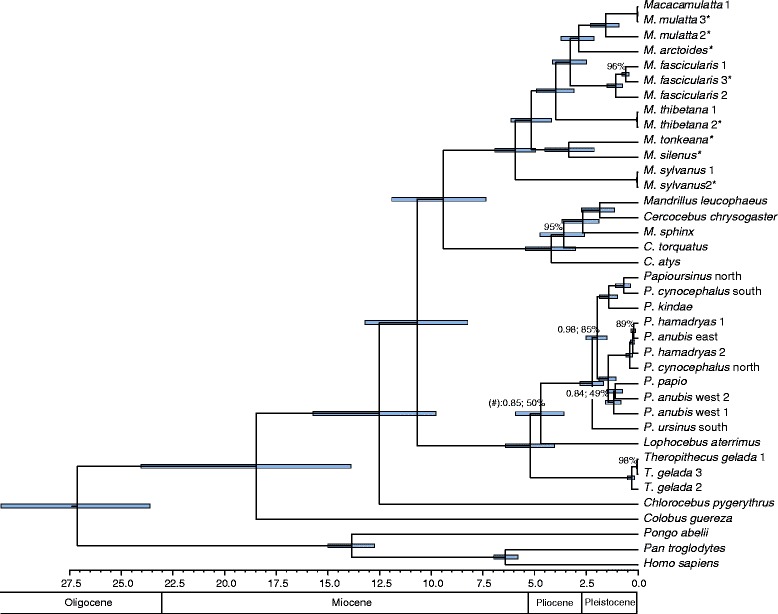
**Ultrametric tree of the Papionini and outgroup taxa as inferred from mtDNA dataset 1.** Tree topologies as inferred from Bayesian (MrBayes) as well as from ML (RAxML) estimation were identical with one exception: At one node (labelled with #) the ML tree indicates *Lophocebus* as sister lineage to the *Papio* + *Theropithecus* clade (not depicted). All unlabelled branches show ML BP of 100% and Bayesian PP of 1.0. Values below are indicated at respective nodes. Blue bars indicate 95% credibility intervals of divergence ages. Time scale shows million years before present. For information about taxa and samples see Additional file 7: Table S2. * = sequences were newly generated in this study.

**Figure 2:**
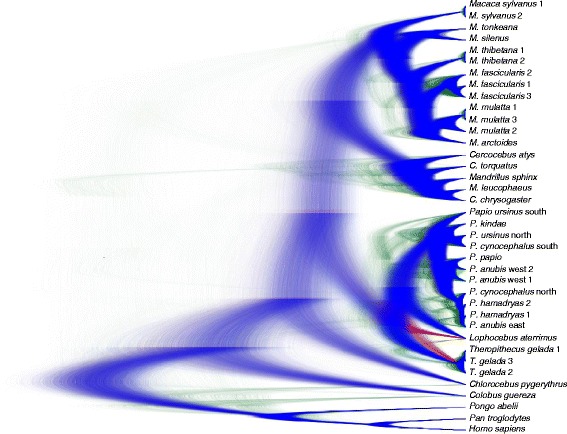
**Densitree showing the posterior probability of 25,000 trees taken from the Bayesian divergence age analysis in BEAST.** Blue represents the most frequent tree topology, red represents the second and green the third most frequent topology.

**Figure 3:**
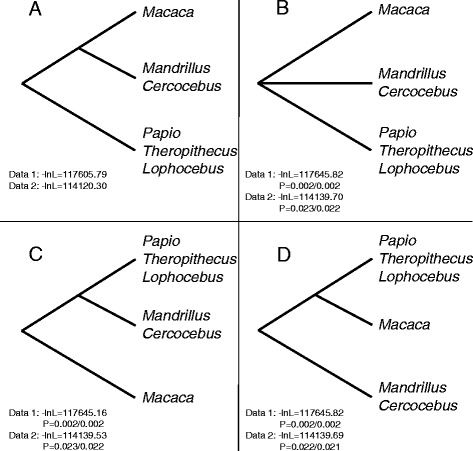
**Tree topologies that were tested in the alternative tree topology test.** Tree **A** represents the most probable topology, whereas **B**, **C** and **D** were significantly rejected. Log-likelihood and P values for each tree topology are given for dataset 1 and 2, respectively. First and second P values resulted from the Kishino-Hasegawa and the Shimodaira-Hasegawa tests, respectively.

The phylogenies as obtained from maximum-likelihood (ML) and Bayesian analyses are mainly identical and most branching patterns are strongly supported (Figure 1). Likewise, the Densitree [62] depicting the posterior distribution of the 25,000 trees as inferred from the Bayesian divergence age analysis in BEAST suggests the most frequent tree topology to be identical to that obtained from ML and Bayesian analyses (Figure 2). According to divergence age estimations using autocorrelated and uncorrelated clock models, the Old World monkeys (Cercopithecoidea) diverged from the Hominoidea between 24 and 27 Ma (for 95% credibility intervals see Additional file 2: Table S1). In the Early Miocene, the two subfamilies of the Cercopithecidae, Colobinae and Cercopithecinae, separated, and the latter subfamily further split into Cercopithecini and Papionini between 11 and 16 Ma. Our analysis revealed three major clades within the Papionini which diverged 9–13 Ma. Interestingly, the *Mandrillus* + *Cercocebus* clade forms a sister lineage to *Macaca* (ML bootstrap value [BP]: 100%; Bayesian posterior probability [PP]: 1.0) and does not cluster with the second major African papionin clade comprising *Papio, Lophocebus* and *Theropithecus* (BP: 100%; PP: 1.0). Since *Mandrillus* and *Cercocebus* show a shift in A/C content similar to macaques (Additional file 3: Figure S1), which could lead to an artificial clustering [63], we repeated our analysis with a modified dataset (dataset 2) that corrects for this shift. Accordingly in this second alignment we masked positions that contain both an Adenin and Cytosin with an “M”. The resulting overall branching pattern and specifically the phylogenetic position of the *Mandrillus* + *Cercocebus* clade among papionins were identical to those obtained from the original dataset (Additional file 4: Figure S2). To further test for alternative positions of the *Mandrillus* + *Cercocebus* clade among papionins, we performed alternative tree topology tests, which revealed that all alternative options are statistically rejected (Figure 3).

Within the *Mandrillus* + *Cercocebus* clade, members of both genera do not form reciprocally monophyletic clades. In dataset 1 *C. atys* is the first lineage to split off (4.2-4.9 Ma) followed by *C. torquatus* (3.6-4.3 Ma), while *M. sphinx* represents a sister lineage to *C. chrysogaster* and *M. leucophaeus* (BP: 100%; PP: 1.0) which separated from them 2.7-3.4 Ma. The latter two diverged 1.9-2.6 Ma. The Bayesian analysis of dataset 2 shows the same topology, but partly with low support (PP: 0.56) while the ML analysis of dataset 2 suggests a possible clade consisting of *C. atys* and *C. torquatus* which, however, is only weakly supported (BP: 49%) (Additional file 4: Figure S2).

Within the second African papionin clade, the branching pattern among the three genera *Papio*, *Theropithecus* and *Lophocebus* is not well resolved. While in the Bayesian analysis of the original dataset, *Theropithecus* is suggested as the first lineage to diverge (PP: 0.85), ML analysis of dataset 1, as well as ML and Bayesian analyses of dataset 2 indicates a *Theropithecus* + *Papio* clade to the exclusion of *Lophocebus*. Node supports for respective branching patterns are low (dataset 1, BP: 50%; dataset 2, PP: 0.89; BP: 83%). Similarly, the Densitree indicates *Lophocebus* + *Papio* as the most frequent clade, while the second most frequent clade is formed by *Theropithecus* and *Papio*. Estimated divergence ages suggest that respective splitting events occurred during a short time period around 5 Ma. Among *Papio* representatives the tree topology is identical and divergence ages are similar as previously reported [54], depicting paraphyletic relationships in *P. ursinus, P. cynocephalus* and *P. hamadryas*, and polyphyletic relationships in *P. anubis*. According to estimated divergence ages, splitting events within *Papio* started around 2 Ma.

Among macaques, *Macaca sylvanus* diverged first, 5.9-6.3 Ma. Subsequently the Asian macaques radiated and successively split into the six Asian species groups. The *M. silenus* + *M. tonkeana* (*M. tonkeana* as representative of the Sulawesi group) clade separated from the remaining macaques between 5.2-5.9 Ma and further segregated into two species groups (3.2-4.6 Ma). Among the remaining macaques, *M. thibetana* (as representative of the *M. sinica* group) diverged between 3.9-5.0 Ma from a *M. fascicularis* + *M. arctoides* + *M. mulatta* clade. Within the latter, *M. fascicularis* split off first (3.2-4.6 Ma) whereas *M. arctoides* separated from the *M. mulatta* clade slightly later (2.7-4.3 Ma). Within *M. fascicluaris* and *M. mulatta* we found relatively ancient splitting events of 1.1-2.2 Ma and 1.4-2.9 Ma.

## Discussion

The application of complete mtDNA genome sequences revealed highly supported branching patterns for most of the investigated papionin lineages. The mtDNA gene tree as well as estimated divergence ages are broadly consistent with those reported in previous studies, but also show some remarkable, but not unexpected discordances to recent nDNA studies [15,19,20,54,64,65].

The major findings of our analysis are: 1) a sister grouping of *Macaca* and the *Mandrillus* + *Cercocebus* clade, 2) paraphyly within the *Mandrillus* + *Cercocebus* clade, 3) unresolved relationships among *Papio*, *Lophocebus* and *Theropithecus*, and 4) similar divergence ages among *Macaca* species groups and papioninan genera. Furthermore, our phylogenetic reconstruction reveals highly supported branching patterns among the seven *Macaca* species groups, which are largely in agreement with most previous studies (e.g. [15,37,66]). The only exception is the phylogenetic position of *M. arctoides*, which is here strongly supported as the sister lineage to the *M. mulatta* group. This finding is not surprising given the evidence that *M. arctoides* is the result of hybridization between ancestral forms of the *M. sinica* and *M. mulatta* groups [37,66].

Divergence dates are mostly consistent regardless of the software (BEAST or PhyloBayes) and clock model (auto-correlated or uncorrelated) that were applied (Additional file 2: Table S1, Additional file 5: Figure S3, Additional file 6: Figure S4). Our estimation indicates a separation of African and Asian macaques around 6 Ma which is in line with Alba et al. [27], who, based on fossil data, proposed a macaque dispersal from Africa into Eurasia by the Late Miocene (5.3-5.9 Ma). Generally, our divergence age estimations reveal a stepwise but rapid radiation of macaque species groups between 5.9 and 2.7 Ma in Asia, which is in agreement with the appearance of the earliest *Macaca*-like fossil in Asia which was found in the Yushe Basin (China) from about 4 Ma [27]. At that time two of the six main lineages of Asian macaques were already established as indicated by our divergence age estimations. To further test possible dispersal scenarios in Southeast Asia and especially in Sundaland additional taxa of the species groups from different locations have to be included in future analyses.

We found the *Mandrillus* + *Cercocebus* clade to be more closely related to the macaques than to other African Papionina, a pattern also reported by Finstermeier et al. [19] and Pozzi et al. [20]. However, in contrast to Finstermeier et al. [19] alternative tree topology tests with our data were clearly rejected (Figure 3), which most likely can be explained by the increased taxon sampling in our study (33 sequences this study, 11 sequences in Finstermeier et al. [19]), because it is known to reduce phylogenetic error [67–70]. Moreover, since we controlled for the observed shift in A/C content, the *Mandrillus* + *Cercocebus* clade might be indeed more closely related to *Macaca* than to the other African papionins, at least if we consider mtDNA. This finding, however, is contradictory to relationships based on recent nuclear studies, which found the Macacina and Papionina to be reciprocally monophyletic [15,18]. Perelman et al. [15] found this branching pattern in a concatenated dataset of 54 nDNA loci (BP: 100%) as well as in six separately analysed subsets, of which four are similarly highly supported (BP: 97-100%). Likewise, the presence/absence pattern of Alu integrations revealed no conflicting integrations, suggesting reciprocal monophyly of both clades [18] and Springer et al. [71], analysing a combined dataset of mtDNA and nDNA sequences, found the same pattern. Interestingly, comparative morphological studies investigating postcranial traits of African Papionina (*Mandrillus*, *Cercocebus*, *Lophocebus* and *Papio*) and one species of *Macaca* (*M. nemestrina*) suggest some similarities between *Mandrillus* + *Cercocebus* and the macaque [45,55]. However, since only one macaque species was included in the analysis, results concerning the relationship of *Mandrillus* + *Cercocebus* to *Macaca* have to be considered with caution. The question is whether the similarities between *Mandrillus*, *Cercocebus* and *M. nemestrina* are due to the plesiomorphy of the traits as suggested by Fleagle & McGraw [45,55] or whether they result from convergent adaptations to similar ecological niches since *Mandrillus*, *Cercocebus* and *M. nemestrina* are predominantly forest dwelling terrestrial primates [72,73]. Given that nDNA phylogenies (e.g. [15]) may reflect the true species relationships more reliably than mtDNA phylogenies with *Macaca* being basal to the Papionina, we would assume that morphological similarities result from convergent adaptation. In contrast, the present mtDNA phylogeny would rather accord to the assumption that the shared morphological features are primitive.

Inconsistencies of mitochondrial and nuclear phylogenies are often explained by incomplete lineage sorting or ancient hybridization [5,19,37,59,60,74,75]. At the moment, we cannot determine if one or both phenomena affected the suggested phylogenetic relationships. A possible scenario based on hybridization could be that ancestral representatives of the *Mandrillus* + *Cercocebus* clade were indeed more closely related to ancestral macaques, but were later introgressed by an ancestor of the *Papio* + *Theropithecus* + *Lophocebus* clade, resulting in nuclear swamping. Hybridization seems to be common among extant papioninan taxa, even between genera [11,12,76,77]. It is therefore likely that hybridization and introgression also occurred among the ancestral papioninan lineages which lead to the observed incongruence between nDNA and mtDNA phylogenies. However, as mentioned above, incomplete sorting of mitochondrial lineages in these taxa is also a plausible explanation for the observed relationships.

Our mtDNA genome tree revealed paraphyletic relationships of *Mandrillus* and *Cercocebus* taxa, which is again contradictory to nDNA studies that suggest both genera to be reciprocally monophyletic [14,15]. As our data show, *M. leucophaeus* clusters with *C. chrysogaster* and *M. sphinx* is indicated as sister lineage to both to the exclusion of *C. torquatus* and *C. atys*. Again, ancient hybridization and incomplete lineage sorting cannot be excluded as having affected this branching pattern. However, since the species identification of the herein used *C. torquatus* sample is questionable (originally identified as *Lophocebus albigena* [78]), our results have to be regarded as preliminary and at the moment any further discussion of possible phylogeographic scenarios would remain highly speculative. Interestingly, however, the sister relationship of *C. chrysogaster* to *M. leucophaeus* is consistent with Kingdon’s [79] p.46 observation that *C. chrysogaster* is morphologically “the most drill-like of the drill-mangabeys”. On the other hand, Kingdon’s suggestion has not been held up by several other studies, which find *C. torquatus* to be the most primitive and *Mandrillus*-like mangabey [14,45,46,55,72]. Comprehensive sampling of mangabeys with reliable information on their geographic provenance is required to further elucidate relationships within the *Mandrillus* + *Cercocebus* clade.

Relationships among *Papio*, *Theropithecus* and *Lophocebus* have been analysed in several studies, but differed depending on the markers that were applied. Chatterjee at al. [56] investigated seven mitochondrial genes and found *Theropithecus* clustering with *Lophocebus* to the exclusion of *Papio* while Finstermeier et al. [19] showed a closer, but only weakly supported mtDNA genome affiliation of *Papio* to *Theropithecus*; Pozzi et al. [20] were also not able to resolve these relationships. Likewise, while we found *Theropithecus* split off first in the Bayesian analysis of the original dataset, ML analysis as well as both, Bayesian and ML estimations of dataset 2 suggested *Lophocebus* in the basal position. For both datasets, support values for respective branching patterns are low and estimated divergence ages among the three genera indicate a rapid radiation around 5 Ma. Also in the Densitree, different branching patterns are depicted. Accordingly, the present data are probably not sufficient to resolve the branching pattern. On the other hand, nDNA sequence data revealed a more consistent picture by placing *Lophocebus* with *Papio* to the exclusion of *Theropithecus* [14,15,48,56,71]. Not surprisingly, morphological (i.e., craniodental) data are congruent with these molecular studies when allometry is properly accounted [80,81]. Guevara & Steiper [14] stated that the basal position of *Theropithecus* is plausible given that known fossils [82] of the genus are considerably older (~4.0 Ma) than those of *Papio* (~2.5 Ma) and *Lophocebus* (~2.0 Ma). It has been shown that an increased sampling of more individuals per species may help to resolve phylogenies with short internodes, but nevertheless an increased sampling will not improve the phylogenies when hybridisation has confounded it [14,74].

The initial radiation within the Papionini into the three main lineages 1) *Papio*, *Theropithecus* and *Lophocebus*, 2) *Mandrillus* and *Cercocebus*, and 3) *Macaca* took place during the Late Miocene. Within these three clades, further differentiation events occurred on similar time scales (*Theropithecus* – *Lophocebus – Papio*: 5–6 Ma; *Mandrillus* – *Cercocebus*: 4–5 Ma; *Macaca*: 5–6 Ma). (Figure 1, Additional file 2: Table S1, Additional file 4: Figure S2). This means that, although macaques seem morphologically not as diverse as their African sister taxa [23,35,83], the mitochondrial heterogeneity among species groups is at least as high as among the African papionin genera. Comparing our mtDNA divergence ages with those inferred from nDNA data (e.g. [15]) we find that those splits slightly differ but tend to be in the same range (Additional file 2: Table S1). We therefore can assume nuclear heterogeneity among *Macaca* species groups and Papionina genera to be also in a similar range.

Given the equally long independent evolutionary histories of macaque species groups and Papionina genera the question of whether the species groups represent rather distinct genera or whether the two main African Papionina clades constitute only two genera (*Papio* and *Cercocebus*) with diverse species groups seems a subject for debate. However, due to morphological similarities of the macaque taxa and the morphological differences between the African genera, a reorganisation of their taxonomic ranks based on time depths as proposed by Goodman [84] and Groves [23,85] seems not to be justified at the moment.

## Conclusion

By analysing complete mtDNA genomes of all papionin genera (with the exception of *Rungwecebus*) we obtained well-resolved phylogenetic relationships and higher support values than inferred from shorter mtDNA fragments. Our estimated divergence ages are similar to those of other studies but credibility intervals are narrowed down due to the application of complete mtDNA genome sequences. Including an increased number of papionin samples led to a different tree topology concerning the phylogenetic position of the *Mandrillus* + *Cercocebus* clade among papionins, which is in stark contrast to previous nDNA studies, indicating that ancient introgression or incomplete lineage sorting may have played a role here. However, which of the two processes led to these contradictions cannot be determined here since we analysed only the maternal lineage of included taxa.

Although the mtDNA tree is just a single gene tree, it offers important additional information on the evolutionary history of the Papionini. Future investigations should incorporate a large number of nDNA loci or even complete genome data to possibly distinguish introgression or incomplete lineage sorting. Furthermore, for a reliable comparative study of mtDNA and nDNA sequences data, respective loci are at best obtained from the same individuals or at least the same species. In addition to nDNA data future studies should also include comprehensive sequence data of the herein unstudied genus *Rungwecebus*. There is also a need to further elucidate intra-generic taxonomy and phylogeny in almost all papionin genera, particularly in *Cercocebus*. Therefore special attention must be paid to the geographic provenance of studied samples.

## Methods

### Sample collection

Blood samples from one individual each of *M. arctoides* (*M. arctoides* group), *M. silenus* (*M. silenus* group), *M. tonkeana* (Sulawesi group), *M. fascicularis* (*M. fascicularis* group) and *M. sylvanus* (*M. sylvanus* group), and two individuals of *M. mulatta* (*M. mulatta* group) were obtained from European zoos, Covance Inc., Münster, Germany and the German Primate Center. All blood samples were taken during routine health checks by experienced veterinarians and not specifically for this study. A fresh tissue sample from a deceased *M. thibetana* (*M. sinica* group) individual was obtained from the Strasbourg Primate Center. Sample collection was approved by the Animal Welfare Body of the German Primate Center and adhered to the American Society of Primatologists Principles for the Ethical Treatment of Non-Human Primates (see www.asp.org/society/resolutions/EthicalTreatmentOfNonHumanPrimates.cfm). No animals were sacrificed for this study.

### Laboratory methods

Genomic DNA from blood and tissue samples was extracted using the Qiagen DNeasy Blood & Tissue Kit following the supplier’s recommendations. To minimize the chance of amplifying nuclear mitochondrial-like sequences (numts) [86], two overlapping long-range PCR fragments were generated (8 kb and 10 kb) using primers specifically designed for macaque species groups on the basis of available sequence data in GenBank and the Long Range dNTPack from Roche. Conditions for the long-range PCR amplification comprised a pre-denaturation step at 94°C for 2 min, followed by 40 cycles at 94°C for 1 min, annealing at 60°C for 1 min and extension at 68°C for 20 min. At the end a final extension step at 68°C for 30 min was added. PCR products were visualized on 1% agarose gel and extracted with the Qiagen PCR purification Kit. Obtained long-range fragments were used as template for nested PCRs to generate products of 1.0 to 1.2 kb. Respective primers are available from the authors upon request. PCR conditions for nested PCRs comprised a pre-denaturation step at 94°C for 2 min, followed by 40 cycles each with denaturation at 94°C for 1 min, annealing at 60°C for 1 min and extension at 72°C for 1.5 min, and terminating with a final extension step at 72°C for 5 min. PCR products were again checked on 1% agarose gels, and subsequently extracted and sequenced on an ABI 3130*xL* sequencer using the BigDye Terminator Cycle Sequencing Kit (Applied Biosystems) and the amplification primers. DNA extraction, PCR set-up, gel extraction and sequencing were performed in separate laboratories. Genome sequences were assembled with SeaView 4.4.0. [87] and annotation was conducted with the online program DOGMA [88] and manually checked. Sequences in the overlapping parts of the two long-range PCRs were identical and all protein-coding genes were correctly translated without any premature stop codons, indicating that no numt contamination is present in our data. All sequences were deposited at GenBank (for accession numbers see Additional file 7: Table S2).

### Data analysis

The dataset for the phylogenetic analysis comprised a total of 38 mtDNA genome sequences including 13 macaques representing all seven species groups (2 *M. sylvanus*, 1 *M. silenus*, 1 *M. tonkeana*, 2 *M. thibetana*, 3 *M. mulatta*, 3 *M. fascicularis* and 1 *M. arctoides*), eleven baboons (2 *P. ursinus*, 2 *P. hamadryas*, 3 *P. anubis*, 2 *P. cynocephalus*, 1 *P. kindae* and 1 *P. papio*), three geladas (*T. gelada*), one drill (*M. leucophaeus*), one mandrill (*M. sphinx*), one crested mangabey (*L. aterrimus*), three capped mangabeys (1 *C. chrysogaster*, 1 *C. atys*, 1 *C. torquatus*) and five non-papionin primate species (*Chlorocebus pygerythrus, Colobus guereza, Pongo abelii, Pan troglodytes, Homo sapiens*). Accordingly, *Rungwecebus* was the only missing papionin genus. The identity of the *C. torquatus* individual remained ambiguous. While it was originally assigned to *Lophocebus albigena* [78], BLAST-search revealed that it is 99-100% identical to available mtDNA sequences of *C. torquatus*. For information about GenBank accession numbers and the source of the herein used sequences see Additional file 7: Table S2.

Sequences were aligned with Muscle 3.7 [89] as implemented in SeaView and manually corrected. For phylogenetic tree reconstructions, indels and poorly aligned positions were removed with Gblocks 0.91b [90]. To check for possible shifts in base composition among species, we calculated the base composition for each species using PAUP 4.0b10 [91]. Since we observed a slight shift in A/C content among papionins (Additional file 3: Figure S1) and to test whether this shift might have influenced phylogenetic inferences, we generated a second alignment (dataset 2) in which positions that contained both an Adenin and Cytosin were masked with an “M” (in total 606 positions).

The programs RAxML 0.93 [92] and MrBayes 3.1.2 [93,94] were used for phylogenetic tree reconstructions applying ML and Bayesian algorithms. As substitution models for Bayesian reconstructions we applied the TrN + I + G and GTR + I + G models for datasets 1 and 2, respectively, as they were selected as best-fit models by jModeltest 2.1 [95] under the Bayesian information criterion (BIC) and the Decision Theory Performance-based Selection (DT). In MrBayes we analysed four independent Markov Chain Monte Carlo (MCMC) runs with a default temperature of 0.2. All repetitions were run for 1 million generations with tree and parameter sampling setting in every 100 generations. The first 25% of samples were discarded as burn-in, resulting in 75,001 trees per run. The adequacy of the burn-in and convergence of all parameters was assessed via the uncorrected potential scale reduction factor (PSRF) [96] as calculated by MrBayes and by visual inspection of the trace of the parameters across generations using the software TRACER 1.5 [97]. To check whether posterior clade probabilities were also converging, AWTY [98] was used. Posterior probabilities for each split and a phylogram with mean branch lengths were calculated from the posterior density of trees. Both ML calculations in RAxML were run with the CAT-GTR model and 1,000 rapid bootstrapping replications. Alternative phylogenetic relationships among the three observed major papionin clades were tested with the Kishino-Hasegawa test [99] and Shimodaira-Hasegawa test [100] with full optimisation and 1,000 bootstrap replications in PAUP.

Divergence ages were estimated applying both, uncorrelated and autocorrelated, clock models. To calculate divergence ages with an uncorrelated clock model, we used BEAST 1.6.1 [101,102]. We assumed a relaxed lognormal model of lineage variation and a Birth-Death Process prior for branching rates. In contrast to Finstermeier et al. [19], branching of *Mandrillus* + *Cercocebus* with *Macaca* was not constrained in our study as alternative branching patterns were rejected by alternative tree topology tests.

The following five fossil-based calibration points were applied with a normal distribution prior for respective nodes: The *Homo* – *Pan* split 6.5 Ma with a 95% credibility interval (CI) of 0.5 Ma [103–105]. The split between *Pongo* and the *Homo*-*Pan* lineage at 14.0 Ma (95% CI: 1.0 Ma) [106], the divergence of *Theropithecus* and *Papio* 5.0 Ma (95% CI: 1.5 Ma) [107,108], the split between African and Asian macaques at 5.5 Ma (95% CI: 1.0 Ma) [27,108] and the separation of hominoids and cercopithecoids at 27.5 Ma (95% CI: 3.5 Ma) [109–111].

In total, we ran four replicates in BEAST, each with 25 million generations, and tree and parameter sampling every 1,000 generations. TRACER was applied to assess the adequacy of a 10% burn-in and the convergence. The sampling distributions were combined (25% burn-in) with LogCombiner 1.6.1 and a consensus chronogram with node height distribution was generated and visualized with TreeAnnotator 1.6.1 and FigTree 1.4.0 [112].

To see whether the application of an autocorrelated model instead of an uncorrelated model has an effect on the divergence time estimation we performed Bayesian molecular dating with the software package PhyloBayes 3.3 [113]. The tree topology was fixed using the topology as inferred from MrBayes. Five node ages were fixed by specifying calibration intervals based on the same calibration points and credibility intervals as mention above. In the main program of PhyloBayes (pb) the CAT-GTR model was applied in combination with a log-normal auto-correlated (−ln) [114] relaxed clock model and in a second independent run with an uncorrelated (−ugam) [101] relaxed clock model. We monitored the development of the log-likelihood as a function of time and found it to be stable (to show convergence) after approximately 3000–4000 cycles. Hence, 10,000 cycles were carried out discarding the first 2,500 trees as burn-in. A posterior consensus chronogram was calculated on the remaining 7,500 trees using the post analysis program readpb and was visualized with FigTree.

### Availability of supporting data

The data set supporting the results of this article is available in the Data Dryad repository, DOI: 10.5061/dryad.9tm42.

## Additional files

Additional file 1:
**Original alignment of complete mitochondrial genomes from 38 catarrhine primate taxa.**
Additional file 2: Table S1. Divergence ages among catarrhine primates in Ma (95% credibility intervals) estimated with uncorrelated and autocorrelated relaxed clock models. Additional file 3: Figure S1. Nucleotide composition among Papionini and outroup taxa. Additional file 4: Figure S2. Ultrametric tree of Papionini and outgroup taxa as inferred from dataset 2. Tree topologies as inferred from Bayesian (MrBayes) as well as from ML (RAxML) estimations were mainly identical with some exceptions. All unlabelled branches show ML BP of 100% and Bayesian PP of 1.0. Values below are indicated at respective nodes. Taxa indicated with ^a^ are arranged differently in the ML (RAxML) and Bayesian tree (MrBayes): ((*P. anubis* west2, *P. anubis* west1) *P. papio*); ((*C. torquatus, C. atys*), ((*C. chrysogaster, M. leucophaeus*), *M. sphinx*)). Red ellipse indicates main difference to Figure 1. * = sequences were newly generated in this study. Additional file 5: Figure S3. Tree topology including divergence dates as estimated with an auto-correlated relaxed clock model as implemented in PhyloBayes 3.3. Time scale shows million years before present. * = sequences were newly generated in this study. Additional file 6: Figure S4. Tree topology including divergence dates as estimated with an uncorrelated relaxed clock model as implemented in PhyloBayes 3.3. Time scale shows million years before present. * = sequences were newly generated in this study. Additional file 7: Table S2. Studied species and individuals along with their GenBank accession numbers.
