# Supplementary material for: Mitogenomics of the Old World monkey tribe Papionini
Source: BMC Evol Biol. 2014 Sep 4;14:176. doi: 10.1186/s12862-014-0176-1 (PMC4169223; doi:10.1186/s12862-014-0176-1)
Supplement: Additional file 2: Table S1. — Divergence ages among catarrhine primates in Ma (95% credibility intervals) estimated with uncorrelated and autocorrelated relaxed clock models. [file 12862_2014_176_MOESM2_ESM.docx]

**Table S1.** Divergence ages among catarrhine primates in Ma (95% credibility intervals) estimated with uncorrelated and auto-correlated relaxed clock models based on dataset 1.

|  | uncorrelated (BEAST) | uncorrelated  (PhyloBayes) | autocorrelated (PhyloBayes) |  |
| --- | --- | --- | --- | --- |
| Split | mtDNA divergence ages | mtDNA divergence ages | mtDNA divergence ages | nDNA divergence ages^1^ |
| Cercopithecoidea – Hominoidea | 27.12 (23.62-30.83) | 27.42 (24.23-30.76) | 24.62 (24.02-26.19) | 31.56 (25.66-37.88) |
| *Pongo* – (*Homo* + *Pan*) | 13.86 (12.74-15.01) | 13.82 (13.04-14.90) | 13.75 (13.03-14.88) | 16.52 (13.45-19.68) |
| *Homo* – *Pan* | 6.41 (5.81-6.98) | 6.45 (6.02-6.97) | 6.59 (6.04-6.98) | 6.60 (5.40-7.96) |
| Colobinae – Cercopithecinae | 18.47 (13.89-24.03) | 22.66 (18.21-27.29) | 15.78 (13.00-18.87) | 17.57 (13.88-21.52) |
| Papionini – *Chlorocebus* (Cercopithecini) | 12.51 (9.77-15.72) | 15.58 (12.03-19.69) | 11.50 (9.53-13.68) | 11.50 (9.18-13.85) |
| (*Macaca* + *Mandrillus* + *Cercocebus*) – (*Papio* + *Theropithecus* + *Lophocebus*) | 10.69 (8.24-13.20) | 12.68 (9.83-16.31) | 10.21 (8.69-11.95) | - |
| *Macaca* – remaining Papionini | - | - | - | 8.13 (6.69-9.68) |
| (*Mandrillus* + *Cercocebus*) – (*Papio* + *Lophocebus* + *Theropithecus*) | - | - | - | 6.67 (5.37-8.07) |
| *Macaca* – (*Mandrillus* + *Cercocebus*) | 9.41 (7.34-11.91) | 10.82 (8.29-14.14) | 9.45 (8.23-10.81) | - |
| *Lophocebus –* (*Papio* + *Theropithcus*) | - | - | - | - |
| *Theropithcus –* (*Papio* + *Lophocebus*) | 5.20 (4.04-6.41) | 5.24 (3.79-6.42) | 6.11 (5.28-6.49) | 4.06 (3.36-4.70) |
| *Papio* – *Theropithecus* | - | - | - |  |
| *Papio* – *Lophocebus* | 4.70 (3.59-5.92) | 4.70 (3.33-6.04) | 5.87 (5.06-6.35) | 3.24 (2.46-4.07) |
| *T. gelada* 2 *–* (*T. gelada* 3 + *T. gelada* 1) | 0.30 (0.16-0.50) | 0.30 (0.13-0.69) | 1.93 (0.89-3.13) | - |
| *T. gelada* 3 – *T. gelada* 1 | 0.04 (0.02-0.08) | 0.04 (0.01-0.12) | 0.80 (0.21-1.83) | - |
| *C. atys –* (*C. torquatus* + *C. chrysogaster* + *M. sphinx* + *M. leucophaeus*) | 4.19 (3.02-5.43) | 4.61 (2.85-7.30) | 4.87 (3.39-6.03) | - |
| *C. torquatus –* (*M. sphinx* + *C. chrysogaster* + *M. leucophaeus*) | 3.59 (2.58-4.75) | 3.82 (2.33-6.11) | 4.29 (2.83-5.51) | - |
| *M. sphinx* – (*C. chrysogaster* + *M. leucophaeus*) | 2.67 (1.88-3.67) | 2.74 (1.60-4.56) | 3.36 (2.05-4.52) | - |
| *C. chrysogaster* – *M. leucophaeus* | 1.85 (1.13-2.74) | 1.85 (0.94-3.29) | 2.63 (1.49-3.73) | - |
| *Mandrillus* – *Cercocebus* | - | - | - | 4.85 (3.58-6.23) |
| *M. sylvanus* – Asian macaques | 5.93 (4.95-6.93) | 5.93 (4.84-6.48) | 6.29 (5.77-6.50) | 5.12 (4.27-5.93) |
| (*M. silenus + M. tonkeana*) – remaining macaques | 5.16 (4.18-6.15) | 5.19 (4.13-6.01) | 5.89 (5.30-6.26) | 4.13 (3.26-5.01) |
| *M. silenus* – *M. tonkeana* (Sulawesi macaques) | 3.34 (2.11-4.51) | 3.23 (1.91-4.58) | 4.58 (3.79-5.19) | 3.13 (2.35-3.98) |
| *M. thibetana* – (*M. mulatta* + *M. arctoides* + *M. fascicularis*) | 3.97 (3.09-4.90) | 3.93 (2.90-4.98) | 5.04 (4.41-5.52) | - |
| *M. fascicularis* – (*M. mulatta* + *M. arctoides*) | 3.28 (2.49-4.14) | 3.18 (2.22-4.20) | 4.61 (3.97-5.10) | - |
| *M. fascicularis* 2 – (*M. fascicularis* 1 + *M. fascicularis* 3) | 1.08 (0.76-1.51) | 1.14 (0.62-1.99) | 2.28 (1.61-3.00) | - |
| *M. fascicularis* 1 *–* *M. fascicularis* 3 | 0.60 (0.43-0.77) | 0.65 (0.32-1.26) | 1.12 (0.72-1.61) | - |
| *M. mulatta* – *M. arctoides* | 2.86 (2.14-3.72) | 2.70 (1.78-3.68) | 4.32 (3.67-4.84) | - |
| *M. mulatta* 2 *–* (*M. mulatta* 1 + *M. mulatta* 3) | 1.56 (0.93-2.30) | 1.41 (0.74-2.35) | 2.94 (2.27-3.50) | - |
| *M. mulatta* 1 *– M. mulatta* 3 | 0.02 (0.01-0.05) | 0.02 (0.01-0.06) | 0.09 (0.02-0.30) | - |
| *M. thibetana* 1 *– M. thibetana* 2 | 0.04 (0.01-0.07) | 0.03 (0.01-0.08) | 0.26 (0.04-0.97) | - |
| *M. sylvanus* 1 *– M. sylvanus* 2 | 0.04 (0.02-0.07) | 0.04 (0.01-011) | 0.36 (0.04-1.30) | - |
| (*M. arctoides* + *M. thibetana*) – (*M. fascicularis + M. mulatta*) | - | - | - | 3.53 (2.69-4.47) |
| *M. fascicularis* – *M. mulatta* | - | - | - | 2.77 (1.94-3.67) |
| *M. arctoides* – *M. thibetana* | - | - | - | 2.38 (1.40-3.37) |
| *Papio ursinus* south – remaining baboons | 2.22 (1.67-2.81) | 2.01 (1.30-3.11) | 3.31 (2.51-3.97) | - |
| (*P. ursinus* north + *P. cynocephalus* south + *P. kindae*) – remaining baboons | 1.98 (1.51-2.52) | 1.84 (1.21-2.83) | 3.18 (2.37-3.84) | - |
| *P. kindae* – (*P. ursinus* north + *P. cynocephalus* south) | 1.41 (0.99-1.86) | 1.27 (0.73-2.07) | 2.52 (1.78-3.17) | - |
| *P. ursinus* north – *P. cynocephalus* south | 0.68 (0.37-1.08) | 0.61 (0.23-0.28) | 1.36 (0.83-1.92) | - |
| (*P. anubis* west2 + *P. papio* + *P. anubis* west1) – (*P. anubis* east + *P. hamadryas* + *P. cynocephalus* north) | 1.44 (1.07-1.89) | 1.29 (0.81-2.05) | 2.51 (1.77-3.18) | - |
| (*P. anubis* west2 + *P. papio*) – *P. anubis* west1 | 1.18 (0.82-1.58) | 1.02 (0.60-1.67) | 2.21 (1.52-2.86) | - |
| *P. anubis* west2 – *P. papio* | 1.10 (0.74-1.49) | 0.94 (0.52-1.54) | 2.13 (1.45-2.78) | - |
| (*P. anubis* east + *P. hamadryas* 1 *+ P. hamadryas* 2) – *P. cynocephalus* north | 0.40 (0.26-0.58) | 0.37 (0.20-0.69) | 0.89 (0.46-1.49) | - |
| *P. hamadryas* 2 – (*P. anubis* east + *P. hamadryas* 1) | 0.25 (0.16-0.37) | 0.21 (0.11-0.40) | 0.58 (0.29-1.04) | - |
| *P. anubis* east – *P. hamadryas* 1 | 0.21 (0.12-0.33) | 0.18 (0.09-0.35) | 0.52 (0.25-0.92) | - |
| *P. papio* – (*P. anubis* + *P. hamadryas*) | - | - | - | 1.21 (0.70-1.79) |
| *P. anubis* – *P. hamadryas* | - | - | - | 0.72 (0.35-1.17) |

^1^ nuclear divergence ages from [1] based on 34,927 bp from 54 genes.
