# Supplementary material for: Mitogenomics of the Old World monkey tribe Papionini
Source: BMC Evol Biol. 2014 Sep 4;14:176. doi: 10.1186/s12862-014-0176-1 (PMC4169223; doi:10.1186/s12862-014-0176-1)
Supplement: Additional file 3: Figure S1. — Nucleotide composition among Papionini and outroup taxa. [file 12862_2014_176_MOESM3_ESM.pptx]

## Slide 1
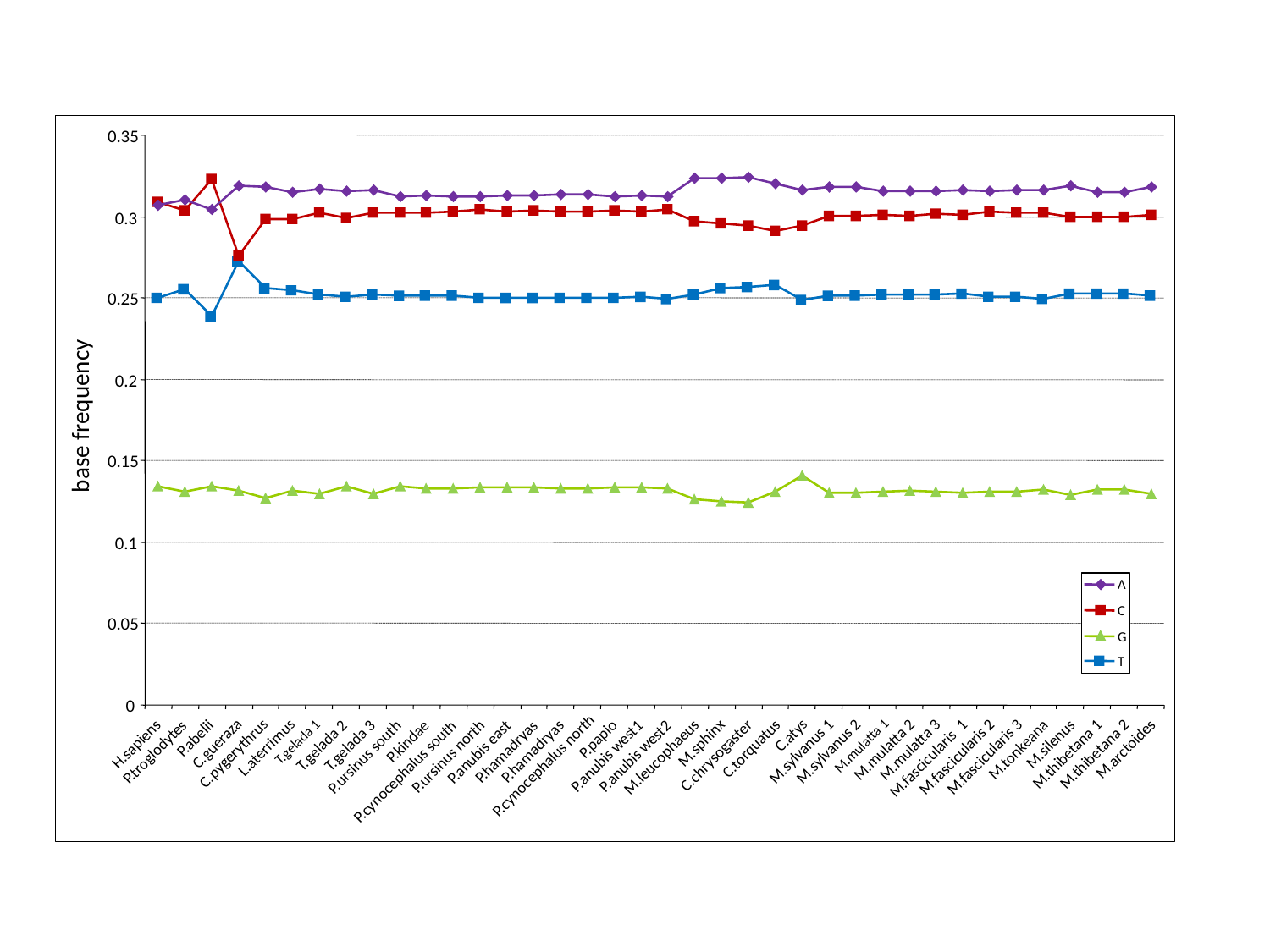

0.35
0.3
0.25
0.2
base frequency
0.15
0.1
A
C
G
T
0.05
0
C.atys
P.abelii
P.papio
P.kindae
M.sphinx
T.gelada 1
C.gueraza
M.silenus
H.sapiens
T.gelada 2
T.gelada 3
M.mulatta 1
L.aterrimus
C.torquatus
M.arctoides
M.mulatta 2
M.mulatta 3
M.tonkeana
P.hamadryas
M.sylvanus 1
M.sylvanus 2
P.hamadryas
P.anubis east
P.troglodytes
C.pygerythrus
M.thibetana 1
M.thibetana 2
C.chrysogaster
P.anubis west1
P.anubis west2
P.ursinus north
P.ursinus south
M.leucophaeus
M.fascicularis 2
M.fascicularis 3
M.fascicularis 1
P.cynocephalus north
P.cynocephalus south
