# Supplementary material for: Mitogenomics of the Old World monkey tribe Papionini
Source: BMC Evol Biol. 2014 Sep 4;14:176. doi: 10.1186/s12862-014-0176-1 (PMC4169223; doi:10.1186/s12862-014-0176-1)
Supplement: Additional file 4: Figure S2. — Ultrametric tree of Papionini and outgroup taxa as inferred from dataset 2. Tree topologies as inferred from Bayesian (MrBayes) as well as from ML (RAxML) estimations were mainly identical with some exceptions. All unlabelled branches show ML BP of 100% and Bayesian PP of 1.0. Values below are indicated at respective nodes. Taxa indicated with a are arranged differently in the ML (RAxML) and Bayesian tree (MrBayes): ((P. anubis west2, P. anubis west1) P. papio); ((C. torquatus, C. atys), ((C. chrysogaster, M. leucophaeus), M. sphinx)). Red ellipse indicates main difference to Figure 1. * = sequences were newly generated in this study. [file 12862_2014_176_MOESM4_ESM.pptx]

## Slide 1
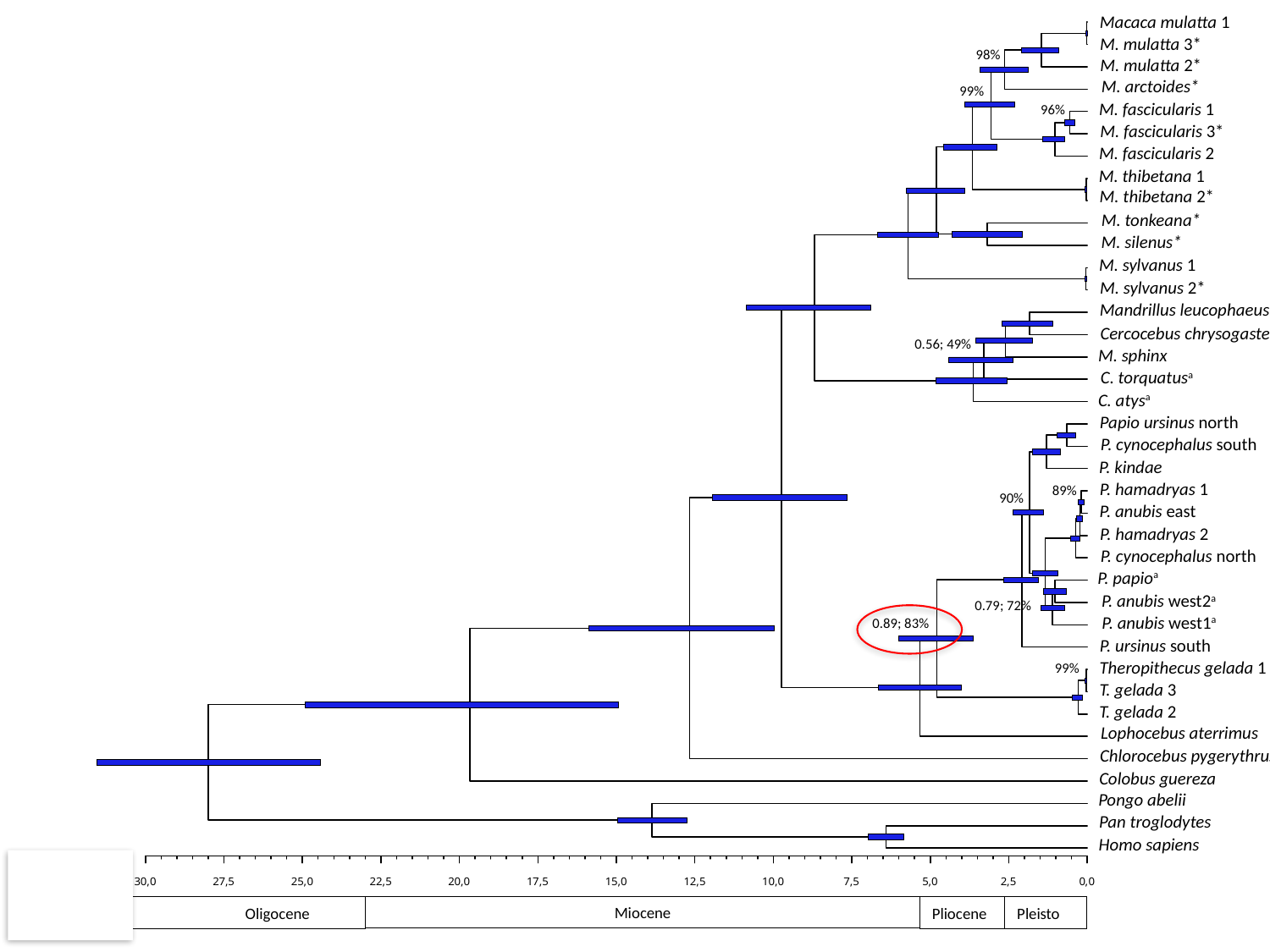

Macaca mulatta 1
M. mulatta 3*
M. mulatta 2*
M. arctoides*
M. fascicularis 1
M. fascicularis 3*
M. fascicularis 2
M. thibetana 1
M. thibetana 2*
M. tonkeana*
M. silenus*
M. sylvanus 1
M. sylvanus 2*
Mandrillus leucophaeus
Cercocebus chrysogaster
M. sphinx
C. torquatusa
C. atysa
Papio ursinus north
P. cynocephalus south
P. kindae
P. hamadryas 1
P. anubis east
P. hamadryas 2
P. cynocephalus north
P. papioa
P. anubis west2a
P. anubis west1a
P. ursinus south
Theropithecus gelada 1
T. gelada 3
T. gelada 2
Lophocebus aterrimus
Chlorocebus pygerythrus
Colobus guereza
Pongo abelii
Pan troglodytes
Homo sapiens
Miocene
Pliocene
Pleisto
 Oligocene
98%
99%
96%
0.56; 49%
89%
90%
0.79; 72%
0.89; 83%
99%
