# Supplementary material for: Mitogenomics of the Old World monkey tribe Papionini
Source: BMC Evol Biol. 2014 Sep 4;14:176. doi: 10.1186/s12862-014-0176-1 (PMC4169223; doi:10.1186/s12862-014-0176-1)
Supplement: Additional file 5: Figure S3. — Tree topology including divergence dates as estimated with an auto-correlated relaxed clock model as implemented in PhyloBayes 3.3. Time scale shows million years before present. * = sequences were newly generated in this study. [file 12862_2014_176_MOESM5_ESM.pptx]

## Slide 1
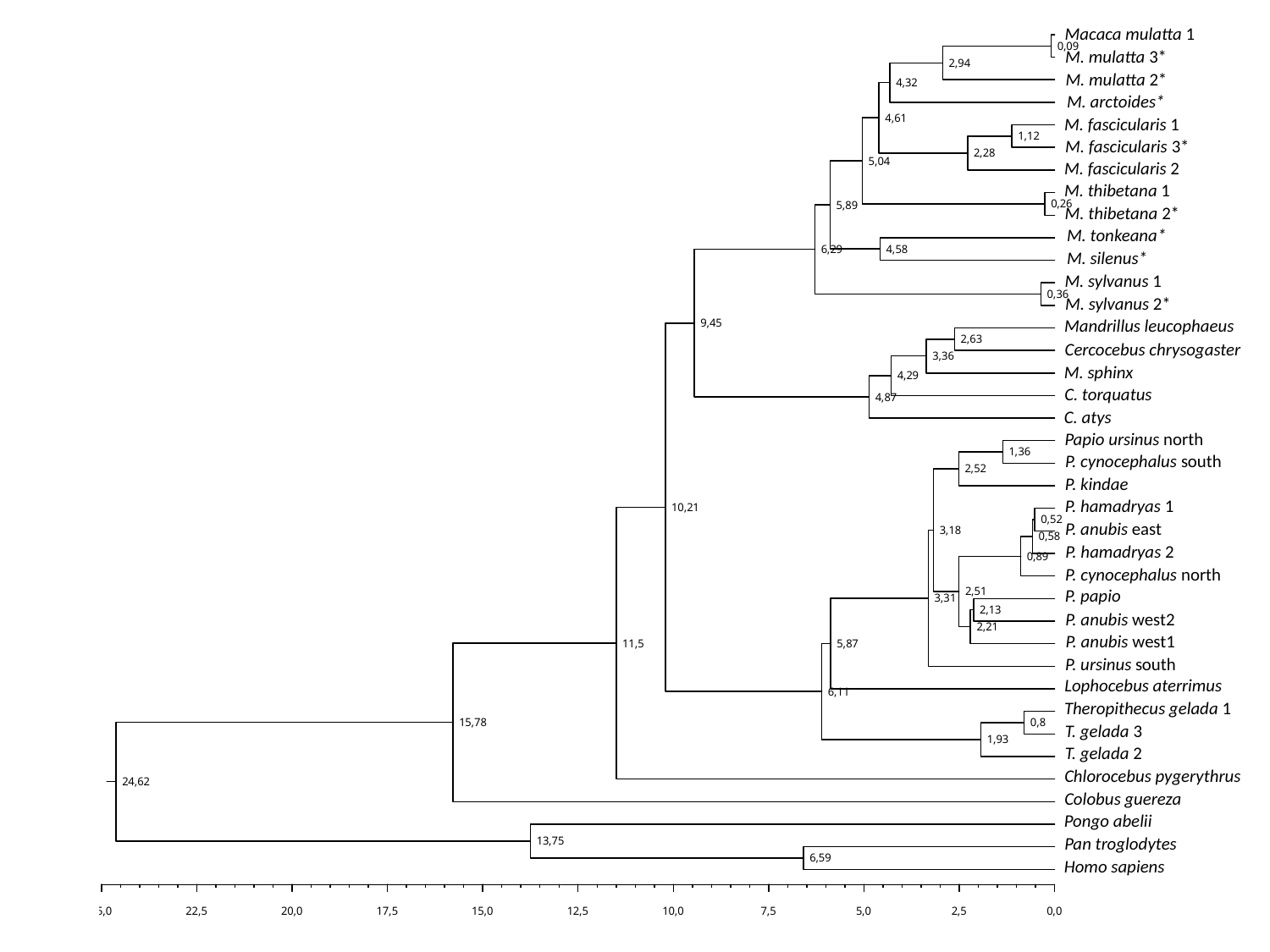

Macaca mulatta 1
M. mulatta 3*
M. mulatta 2*
M. arctoides*
M. fascicularis 1
M. fascicularis 3*
M. fascicularis 2
M. thibetana 1
M. thibetana 2*
M. tonkeana*
M. silenus*
M. sylvanus 1
M. sylvanus 2*
Mandrillus leucophaeus
Cercocebus chrysogaster
M. sphinx
C. torquatus
C. atys
Papio ursinus north
P. cynocephalus south
P. kindae
P. hamadryas 1
P. anubis east
P. hamadryas 2
P. cynocephalus north
P. papio
P. anubis west2
P. anubis west1
P. ursinus south
Lophocebus aterrimus
Theropithecus gelada 1
T. gelada 3
T. gelada 2
Chlorocebus pygerythrus
Colobus guereza
Pongo abelii
Pan troglodytes
Homo sapiens
